# Grape Pulp Fiber as Possible Fining Agents for Red Wine

**DOI:** 10.3390/biom12101519

**Published:** 2022-10-20

**Authors:** Andrea Osete-Alcaraz, Lucía Osete-Alcaraz, Ana Eugenia Ortega-Regules, Ana Belen Bautista-Ortín, Encarna Gómez-Plaza

**Affiliations:** 1 Department of Food Science and Technology, Faculty of Veterinary Sciences, University of Murcia, 30100 Murcia, Spain; 2 Department of Chemical Engineering, Food and Environmental, University of Américas Puebla, Sra. Catarina Mártir, San Andrés Cholula 72810, Mexico

**Keywords:** finning, pulp fiber, cell walls, anthocyanins, tannins, ochratoxin A, histamine

## Abstract

One of the biggest problems with the use of traditional fining agents is that some of them present allergenic characteristics or are not suitable for vegan consumers due to their animal origin. An alternative to these traditional proteinaceous agents could be cell walls from grape pulp. This material could be used to modify the final phenolic concentration of a wine due to its affinity for phenolic compounds. In this study, the ability of freeze-dried grape pulp fiber, rich in pulp cell walls, to act as a fining agent was analyzed in wines from three different varieties: Cabernet Sauvignon, Syrah, and Monastrell. After the use of this material, the wine chromatic characteristics and total tannin concentration were analyzed by spectrophotometric and chromatographic techniques. In addition, the wines were contaminated with ochratoxin A and histamine to check whether this material could also be a tool for removing these wine contaminants. The pulp fiber presented a high capacity to retain phenolic compounds, especially tannins; however, there were differences depending on the studied wine. The largest reduction in tannin concentration after fining was observed when this material was used in Cabernet Sauvignon wines (23%), whereas for Monastrell wines the reduction was lower (18.3%) and even lower for Syrah wines (14.3%). This fining agent also reduced the anthocyanin concentration of the three red wines, although to a lesser extent than the reduction observed for tannins. A really interesting result was that the addition of this fining agent reduced the concentration of ochratoxin A by 50% in all the studied wines.

## 1. Introduction

Clarification or fining consists of adding an adsorbent material to wine for the reduction of undesirable components, with the aim of improving the visual and sensory quality of the resulting wine. One of the most important parameters of the visual quality of wine is its clarity, that is, its degree of turbidity. However, fining is not only focused on reducing turbidity, since other wine components may also be the target of fining agents. One of these targets is the phenolic compounds, since a too high concentration of these compounds, especially tannins, can seriously affect the organoleptic characteristics of the wine, providing excess turbidity, astringency, and bitterness [1].

The origin and nature of the currently used finning agents is varied, although traditionally, the most used fining agents are of animal origin and proteinaceous nature. These fining agents are, essentially, proteins that reduce turbidity by reacting with suspended particles in the wine, generating insoluble aggregates that flocculate and precipitate. They also may react with phenolic compounds forming high molecular weight complexes that end up precipitating [2]. The way in which these proteinaceous agents interact with the phenolic compounds varies depending on the composition of both molecules. For example, in the case of tannins, these fining agents initially interact with tannins by binding them through hydrogen bonds and hydrophobic interactions. After the initial binding, large aggregates are formed by self-association between these protein–tannin complexes, which are insoluble and end up precipitating [2,3,4].

However, the use of proteins of animal origin (especially caseinates and albumins) as fining agents could lead to the presence of allergenic agents in the wine. Another problem derived from the use of these fining agents is that they are rejected by vegan consumers. Therefore, an attempt was made to overcome this problem, by changing the origin of the fining agents for others of plant origin, such as pea or potato proteins [5,6].

Currently, numerous studies have reported the existence of interactions between wine phenolic compounds, such as anthocyanins and tannins, with plant cell walls from different sources such as apple [7], carrot [8], or the skin and pulp of the grapes [9,10,11,12].

The affinity of plant cell walls and grape polyphenols is determined both by the structure and composition of both molecules. However, it also depends on the ratio between their concentrations and the environmental conditions and extrinsic factors in which the interaction occurs, such as, for example, pH, temperature, or ethanol [7,13,14,15,16,17,18].

The use of cell walls from grape skin or purified grape pomace as fining agents, mainly for the reduction of wine phenolic compound and astringency, has already been studied, with positive results when compared with other commercial fining agents. In the study by Jiménez-Martínez et al. [19], the effect of the cell wall material from Monastrell and Cabernet Sauvignon grape pomace on the reduction of the phenolic content of red wines was studied and the results were compared with those obtained with commercial fining agents. In this study, pomace cell walls resulted in a fining effect that exceeded that of most commercial fining agents. The cell wall material significantly reduced the phenolic content of the wines, the reduction varying from 48 to 68% for anthocyanins and from 44 to 64% for tannins, although varietal differences in this regard were observed.

Another beneficial effect of certain fining agents is their ability to bind and eliminate some wine contaminants such as ochratoxin A or biogenic amines.

Ochratoxin A (OTA) is a mycotoxin which is present in grapes, musts, and wines due to fungal contamination with *Aspergillus carbonarius* [20]. Individual operations during winemaking can modify the OTA content in wines, increasing its concentration if long skin maceration time is applied and decreasing during alcoholic and malolactic fermentations [21]. Fining can also reduce the presence of this contaminant. Several oenological fining agents have been tested for reducing the OTA content of wines, although with different degrees of success, depending on the fining agent used, the dosage applied, and the OTA concentration in the wine [22]. The study by Jimenez-Martinez et al. [23] showed that commercial fining agents such as gelatin, egg albumin, or pea protein had a certain OTA retention capacity (approximately 20%) but the purified grape pomace could retain 54–57% of the OTA content in the analyzed wines. Mannoproteins and other byproducts derived from yeasts have also been shown to reduce the concentration of OTA in wines and grape juices [20,24,25].

Biogenic amines are low molecular weight organic compounds. They appeared in wines by microbiological decarboxylation of their amino acid precursors during fermentation, aging or storage [26]. The five biogenic amines most frequently found in wines are: histamine, tyramine, putrescine, cadaverine, and phenylethylamine. Their individual concentrations in wines can vary between 0.8 and 47.3 mg/L [27] and their total quantity varying between 25.2 and 96.8 mg/L [28]. Biogenic amine can induce adverse reactions in sensitive consumers at high concentrations. Once they are present in the wine, fining is the best oenological treatment to decrease their content. Some studies have suggested that, among these oenological fining agents, bentonite and pomace fibers seems to be the most effective at decreasing the content of biogenic amines in wines [23].

Given the results obtained by purified grape pomace (or fibers) as fining agents to reduce the concentration of phenolic compounds and contaminants in wines, we focused our attention on other materials of enological origin that could have promising characteristics as a fining agent. This material is the pulp fiber (rich in pulp cell walls) contained in the pre-fermentative lees, a byproduct of winemaking. According to the study by Bindon et al. [29], the pulp cell walls bind a greater number of tannins than those of the skin, the key structural differences between both cell wall types being the higher endogenous concentration of tightly bound tannins, Klason lignin, and lower amounts of protein in skin cell walls that lead to a reduce flexibility and porosity of these cell walls as compared to the pulp cell walls.

Therefore, the objective of this study is to analyze whether these pulp cell walls present in the purified fiber from pre-fermentative lees could act, similarly to grape skin cell walls and pomace fiber, as a fining agent, studying how the addition of this material affects the phenolic and chromatic composition of wines from three different red varieties: Cabernet Sauvignon, Monastrell, and Syrah. Moreover, their effect on reducing the concentration of two potential wine contaminants (histamine and OTA) was also studied.

## 2. Materials and Methods

### 2.1. Obtention of Pulp Fiber

Red grapes of the Monastrell variety harvested at technological maturity (13.5° Baume) were used for obtaining the pulp fiber, since this material was shown to have capacity for modifying the final phenolic composition of red wine [30]. To obtain this material, grapes were crushed and destemmed and the must was separated from skin and seeds by pressing, using a 75 L pneumatic press. A clarifying enzyme (Enozym Lux, Agrovin, Spain) was added at a dose of 3 mL/hL to the liquid must to accelerate the settling process [30]. Once the enzyme was added, the must was left to settle for 24 h at 10 °C. After this time, the precipitated plant material was collected and centrifuged at 1537× *g* for 5 min and the supernatant was eliminated. Then, the recovered plant material, mainly composed of pulp cell walls and almost free of yeasts (the entire process was done during the pre-fermentative step of vinification), was washed several times with water until the elimination of sugars, frozen, lyophilized, and treated in a mortar until obtaining a fine powder. The composition of the fine powder was analyzed following the assays described in the studies by Apolinar-Valiente et al. [31,32].

### 2.2. Winemaking and Finning

Red grapes of the Monastrell, Syrah, and Cabernet Sauvignon varieties (separately) were crushed and destemmed. All the micro-vinifications for each grape variety were carried out in triplicate in 10 L tanks using 9 kg of grapes. The skin maceration lasted seven days. At the end of the alcoholic fermentation, the wines were racked.

The finning process was carried out in 12 bottles containing 50 mL of wine from each grape variety. Two parallel tests were carried out, one for spectrophotometric and chromatographic analyses, to analyze the phenolic composition of the wine after the fining, and the other to determine the concentration of ochratoxin A and histamine after the previous addition of these wine contaminants. The wines were spiked with histamine to a final concentration of 65 mg/L and ochratoxin A at a final concentration of 9 μg/L and six bottles for each trial were prepared (three of them were added with 0.5 g of the freeze-dried pulp fiber, and three of them were used as controls). The fibers were left for a contact time of 7 days, after which the analyses of the different wines were carried out.

### 2.3. Chromatic Analysis

The color intensity was calculated as the sum of the absorbance at 620, 520, and 420 nm. Total anthocyaninand polymeric anthocyanins concentration were determined by the method described by Ho et al. [33]. The total polyphenol index (TPI) was determined by measuring the absorbance at 280 nm of a wine sample diluted 1:100. Finally, total tannin concentration was calculated using the methylcellulose precipitation method [34].

### 2.4. Tannin Analysis by Phloroglucinolysis

The concentration and composition of the tannins were determined through their reaction with the phloroglucinol reagent, followed by analysis by high performance liquid chromatography, carried out with the method described by Osete-Alcaraz et al. [35].

### 2.5. Analysis of Ochratoxin A by HPLC

The method used to determine the concentration of OTA in the different wines was described by Castellari et al. [36]. First, 10 mL of the samples was adjusted to pH 7.8 with NaOH (2 M) and was diluted with 10 mL of phosphate buffer saline (PBS). The adjusted and diluted samples were centrifuged (5 min at 282× *g*). Ochraprep immunoaffinity columns (R-Biopharm Rhone) were used to clean up the sample, which was injected directly on the immunoaffinity column at a flowrate of about 2–3 mL/min using a vacuum manifold. The column was washed with 20 mL PBS (flow: 5 mL/min) and dried.

A desorption solution (acetic acid 2% in methanol 98%) was used to elute the OTA through the column and then it was collected in an amber vial. To complete the elution, 1.5 mL of ultrapure water were passed.

OTA was quantified by HPLC with fluorescence detection using a Waters liquid chromatograph 2695 (Waters, Milford, MA, USA) equipped with a Waters 2475 fluorescence detector. Chromatographic separations were performed on a Cortecs C18 column, 2.7 μm size particle, 4.6 mm × 75 mm (Waters Milford, MA, USA). The isocratic method used a mobile phase consisting of a mixture of acetonitrile 51%, water 47%, and acetic acid 2% (*v*/*v*/*v*) eluted at a flow rate of 1.0 mL/min at 35 °C.

Finally, the detection was carried out at an excitation wavelength of 333 nm and an emission wavelength of 443 nm. For the quantification, a calibration curve was made for a known amount of the pure standard.

### 2.6. Analysis of Biogenic Amines by HPLC

The method used in this study was developed by Gómez-Alonso et al. [37] and extensively described by Jiménez-Martínez et al. [23].

### 2.7. Statistical Data Treatment

The statistical analysis of the results was carried out using the statistical package Statgraphics Centurion. An analysis of variance (ANOVA) was carried out to determine differences among samples and when there were significant differences, a Test LSD was used to separate the means with a confidence level of 95%.

## 3. Results

### 3.1. Pulp Fiber Composition

Table 1 shows the composition of the grape pulp fiber after the freeze-drying and grinding process. No further purification was done with this material, such that it was mainly composed of pulp material with very little participation of yeast cells since all the recovery process was carried out before the addition of yeasts and the beginning of alcoholic fermentation. The concentration of phenolic compounds in this fiber of the pulp is much lower than that found in grape pomace [19], an expected result since the pulp has, in general, less concentration of phenolic compounds. In addition, this material, due to the way in which it was extracted [30], has barely been in contact with the phenolic compounds from the skins and/or seeds, since it was extracted just after grape crushing and before any maceration took place.

The analysis shows that the protein content is similar to that found in purified skin grape pomace [19]. These authors reported that the amount of protein in Cabernet Sauvignon grape skin pomace was 52.8 mg protein/g pomace, lower than that found in Syrah skin pomace (62.7 mg protein/g pomace) and higher than that found in the Monastrell (40.8 mg protein/g pomace) and Macabeo (48.5 mg protein/g pomace) varieties. The amount of protein in a material that is going to be used as a fining agent will largely determine the interactions with compounds such as tannins. Numerous studies have shown that, in must/wine, the content of proteins from both cell walls [13] and pathogenesis-related proteins [38,39] systematically reduces the tannin content of wines via precipitation. The interaction with polyphenols may be dependent not only on total protein concentration in cell walls, but also the degree to which that protein is solubilized during fermentation/crushing [13]. Due to this ability to interact with certain phenolic compounds, most traditional fining agents are of protein origin.

Another important component when it comes to interacting with phenolic compounds is the amount of pectins that the cell wall contains. We estimated this amount by measuring the concentration of uronic acids (UA) as shown in Table 1. There are numerous studies that show that, among the polysaccharides that make up plant cell walls, pectins are the ones that show the greatest affinity to polyphenols [11,12,13,15], followed by xyloglucans and cellulose. In the grape pulp fiber, the amount of UA was much lower than that observed in purified skin grape pomace [19] or in purified pulp cell walls from different varieties of red grapes [40]. This could probably be due to the action of the enzyme that was used in the settling step of the vinification process (Enozym Lux, a mixture of pectin lyase and polygalacturonase). This enzyme has been shown, in previous studies, to have a high capacity to degrade cell wall pectins and produce a large quantity of soluble low molecular weight polysaccharides [11,12,41]. In the study by Bindon et al. [9], they showed that the use of a pectinolytic enzyme was capable of solubilizing a high concentration of galacturonic acid, rhamnose, and arabinose, both in cell walls from the grape pulp and skin, this being indicative of the depectination that the cell walls have suffered after the enzymatic treatment. Although this reduction in UA may imply a reduction in their ability to interact with phenolic compounds, we should not forget that studies have demonstrated how the action of the enzyme may create porosity (holes) in the cell wall in which these phenolic compounds can be retained [42].

Finally, regarding cellulosic (CS) and non-cellulosic glucose (NCS), their concentration in the fiber of grape pulp was slightly lower than those reported in the studies by Ortega-Regules et al. [40].

### 3.2. Chromatic Characteristics of the Wines

Table 2 shows how the phenolic composition and chromatic parameters of the wines of the Monastrell, Syrah, and Cabernet Sauvignon varieties are modified after pulp fiber contact.

One of the problems derived from the use of fining agents is that they may affect the wine chromatic characteristics, since the chemical characteristics of phenolics makes them prone to interact with the fining agents, so when they are added to a red wine, not only part of the astringent tannins, the target compounds of the treatment, could be eliminated, but also other important compounds such as anthocyanins, which could negatively affect the wine color. It has been systematically shown that the use of different fining agents has a negative impact on the color of red wines by reducing wine anthocyanins [19,43,44]. This reduction has been described for gelatin [19,43,44], mannoproteins [45], vegetable proteins, and grape pomace [19].

Our results when working with pulp fiber showed that this fining agent reduced the concentration of total anthocyanins of the three wines (Monastrell: 12.3%, Syrah: 11.3%, and Cabernet Sauvignon: 12.5%). It also reduced the concentration of polymeric anthocyanins (Monastrell: 12%, Syrah: 13.1%, and Cabernet Sauvignon: 16%). This loss of anthocyanins, as expected, led to a reduction in wine color intensity (Monastrell: 14.6%, Syrah: 14.2%, and Cabernet Sauvignon: 17.5%). It can be seen that the loss of anthocyanins and color intensity is very similar between the Syrah and Monastrell varieties, and slightly higher in Cabernet Sauvignon. This may be due to the fact that the wine from this variety had a higher concentration of polymeric anthocyanins and it has been reported that plant cell walls have more affinity for higher molecular weight or polymerized molecules [11,12], and, as a result, the color of Cabernet Sauvignon wine was the most affected by this fining agent. However, compared to similar studies, the observed reduction in anthocyanin concentration and color is lower than that reported for other commercial fining agents and similar to that observed with vegetable fibers from grape pomace [46].

The measurement of the reduction in tannin concentration using the methylcellulose precipitation method showed that the freeze-dried grape pulp fiber has a high capacity to retain tannins; however, there were differences between the wines from the different varieties. The addition of the fining agent to Cabernet Sauvignon wine led to the greatest reduction in tannin concentration (23%), followed by that observed in Monastrell wine (18.3%) and Syrah wine (14.3%). All these losses are the responsible for the observed decreased in total phenol content.

There are numerous studies that demonstrate the existence of interactions of tannins with grape cell walls, both skin and pulp cell walls. Most of these studies have been carried out in solutions that simulate the composition of wine (model solutions), using purified skin or pulp cell walls and purified tannins. In all of them, it has been shown that the cell walls were capable of removing a significant proportion of tannins from the solution [10,43,44], the percentage of tannin being lost in solution at a much higher rate (more than 50% in most cases) than in our study. This could be probably due to the higher proportion of cell wall per volume unit used in these studies and to the application of agitation during the contact time between phenolic compounds and cell walls. As an example, in the studies by Osete-Alcaraz et al. [11,12], Bindon et al. [47], Le Bourvellece et al. [48], Castro-Lopez et al. [10], and Jiménez-Martínez et al. [19], a tannin reduction between 50% and 67% was reported when the concentration of cell walls in the model solution was of 13 g/L and the samples were subjected to orbital agitation (250–300 rpm) to favor the interaction process.

However, in the study by Jimenez-Martinez et al. [46], using purified grape skin pomace and following the same design used in this study, a reduction of 18.5% in the tannin concentration was reported in a Monastrell wine, a very similar result to that observed for the wine of the same variety in this study (18.3%). Taking into account that in a real vinification, the contact time is usually between 2 to 15 days and continuous agitation is not used in wineries, the reduction in tannin concentration shown in this study could be expected in a real vinification.

As commented previously, Syrah wine showed the lowest reduction in tannin concentration. The different behavior observed between wines from different varieties could be partly explained by the fact that other phenolic compounds, such as anthocyanins, compete with tannins for the plant cell wall binding sites [18], and this fact implies that when cell wall-derived material is used as a fining agent, a large presence of anthocyanins in the wine will hinder, to a certain extent, the interaction with tannins and Syrah wines presented the highest concentration of total anthocyanins and they could compete with wine tannins, decreasing the adsorption of the latter in the fining material.

### 3.3. HPLC Analysis of the Wine Tannin Concentration and Composition as Affected by Fining

The concentration of tannins in the wines was also analyzed by HPLC using the phloroglucinolysis method (Table 3). This chromatographic analysis provides information on the concentration and composition of those depolymerizable wine tannins, that are mainly non-oxidized tannins.

The results for the chromatographic analysis showed that the addition of grape pulp plant material also reduced the concentration of these depolymerizable tannins in the wines, although in the case of the Cabernet Sauvignon wine, the observed decrease with respect to unfined control wine was not significant. The low quantities of depolymerizable tannins in Syrah and Cabernet Sauvignon wines (compared with the concentration measured with the methyl cellulose precipitation method) indicate that a large part of their tannins is under oxidized forms or polymerized with other compounds such as anthocyanins which has been reported to present higher affinity for binding to plant cell walls than simpler, depolymerizable tannins [17,29]. It is clear that, especially in the case of Cabernet Sauvignon wines, the results indicate that most of the adsorbed tannins are non-depolymerizable.

Interestingly, there were no changes in the degree of polymerization of the tannins with the application of the fining agent. Numerous studies have shown that higher molecular weight tannins are the most susceptible to interacting with plant cell walls [10,11,12]. However, Jiménez-Martínez et al. [46] also observed that, in wine samples, neither the contact time nor the dose of purified pomace cell walls modified the mean degree of polymerization of wine tannins.

There was a reduction in the concentration of epigallocatechin and epicatechin gallate in the wine of Monastrell and Syrah varieties, the percentage of reduction being slightly higher for EGC. These results may indicate show that pulp fiber seems to have more affinity for grape skin tannins specially in Monastrell wine.

### 3.4. Removal of OTA and Histamine by Fining

OTA and biogenic amines are two red wine contaminants that generate concern in wineries and consumers. These are generated either during winemaking in alcoholic and malolactic fermentations (biogenic amines) or by fungal contamination of the grape before or after harvest (OTA). One of the techniques that can be carried out in the winery to reduce or eliminate its presence in the wine is fining.

To verify that the pulp fiber was capable of reducing the concentration of these contaminants in wine, an experiment similar to that conducted for the analysis of phenolic compounds was carried out; however, in this experiment, the wines of the three varieties were spiked with histamine (65 mg/L) and OTA (9 μg/L) and their concentration was measured after 7 days of contact with pulp fiber.

The fining agent was able to reduce the concentration of OTA in all the wines (Table 4). The higher reduction was observed in Cabernet Sauvignon wine, with an OTA reduction of 57.8%, followed by Syrah and Monastrell, both with a reduction of 49.4%. OTA is a weak acid that is dissociated at wine pH (3.5) and has a negative charge [49]. Due to these chemical characteristics, OTA has a high reactivity in wine. It has been reported that one of the fining agents with the greatest capacity to eliminate OTA in wine is bentonite [50]. This fining agent presents a negative charge, but due to the presence on the surface of layered aluminum silicate, bentonite has polar terminal regions that are locally positive. OTA also exhibits great reactivity with molecules of a protein nature (positive charge) due to ionic interactions. Jiménez-Martinez et al. [23] observed that the fining agents with the highest OTA retention capacity were bentonite, mannoproteins, and purified grape skin pomace. The latter represented a reduction in the concentration of OTA in a red wine of the Monastrell variety of 54–57%. These authors reported the great capacity of purified skin pomace to retain OTA due to the chemical complexity of the cell walls (polysaccharides, cellulose, hemicellulose and pectins, structural proteins, lignin, and phenols). This same chemical complexity is presented by pulp fiber which, in addition, could also increase the elimination of OTA due to the possible generation of holes in the cell wall structure due to the use of pectinases (polygalacturonase and pectin lyase) to obtain it. The action of the enzyme creates porosity (holes) in the pulp cell wall, increasing the encapsulation of OTA in the cell wall network and reducing much more its content in the wine.

The other contaminant compound investigated in this study is histamine, which is the most common biogenic amine and the one that appears in higher concentrations in wines. Other biogenic amines, such as tyramine, putrescine, cadaverine, or phenylethylamine, are also present in wine, although it has been previously reported that fining is especially effective in removing histamine from wines, while these other biogenic amines are much less affected by fining. In this way, Mayer and Pause [51] reported that among the biogenic amines, histamine was the most susceptible to be removed by the addition of a fining agent such as bentonite. This fact is due to its chemical structure, in which the nitrogen atoms located in the imidazole group are much less basic than that linked to the alkyl carbon (amine group), while in the other biogenic amines, the basicity of the amino group is increased by inductive effect, the alkyl groups may impart electron density to the nitrogen atom [52]. In this way, the rest of biogenic amines are more susceptible to interacting with molecules such as Lewis acids than with the fining agents used.

Pulp fiber reduced the histamine concentration by 8% in Monastrell and Cabernet Sauvignon wines and by 3.4% in the Syrah wine, although in this case, no significant differences were found between the control and the treatment in the Syrah wine. It has been shown that of the currently used fining agents, the one with the greatest capacity to reduce biogenic amines (BA) in must and wine is bentonite [51,53]. Kally and Body-Szalkai [54] observed that in red wines, 80 g/hL of bentonite reduced histamine content by 60%, although Grossman et al. [55] reported that the bentonite is more effective for removing biogenic amines when used in the must instead of wine. In the study by Jimenez-Martinez et al. [23], different types of clarifying agents were studied, such as gelatins, pea proteins, egg albumin, bentonite, and purified grape pomace, looking for the most effective in reducing the concentration of biogenic amines. Of the fining agents used, only bentonite and purified grape pomace were able to reduce histamine by 10%. These results show that plant cell walls, both arising from grape skin or pulp, have a certain ability to remove histamine from wines.

## 4. Conclusions

Pulp fiber has been shown to act very effectively as a fining agent, reducing tannin content in young wines and with the possible advantages of not presenting allergy issues (although no allergic assays have been conducted in this study) and being accepted by vegan consumers. Moreover, this material is a byproduct obtained during the winemaking process, their possible uses in vinifications may contribute to a circular economy. Pulp fiber reduced the concentration of tannins in red wines of different varieties by 15–23%, without affecting their degree of polymerization. The anthocyanin reduction percentage (around 10–12%) was lower than that reported in other fining agents. In addition, this material is capable of a 50% reduction in the concentration of OTA, a contaminant substance that may appear in wines.

## Figures and Tables

**Table 1:** Composition of pulp fiber.

Samples	P	PC	CS	NCS	UA
Pulp fiber	53.29 ± 1.84	18.76 ± 1.30	25.61 ± 0.67	16.11 ± 3.40	3.55 ± 0.64

P, proteins (mg/g of cell wall); PC, phenolic compounds (mg/g of cell wall); CS, cellulosic glucose (mg/g of cell wall); NCS, non-cellulosic glucose (mg/g of cell wall); UA, uronic acids (UA mg/g of cell wall).

**Table 2:** Chromatic characteristics of the wines with and without treatment with the fining agent (pulp fiber).

Samples	CI	TA	TPI	PolA	MCPT
Monastrell					
Control	13.7 ± 0.0 b	606.7 ± 4.1 b	57.8 ± 0.4 b	31.7 ± 0.1 b	1560.2 ± 6.5 b
+Pulp Fiber	11.7 ± 0.0 a	532.3 ± 0.9 a	51.2 ± 0.4 a	27.9 ± 0.1 a	1273.6 ± 39.3 a
Syrah					
Control	20.4 ± 0.3 b	763.8 ± 5.3 b	54.9 ± 0.6 b	66.2 ± 0.2 b	1269.9 ± 54.1 b
+Pulp Fiber	17.5 ± 0.0 a	677.7 ± 4.5 a	50.0 ± 1.0 a	57.5 ± 0.2 a	1088.6 ± 32.9 a
Cabernet Sauvignon					
Control	21.2 ± 0.1 b	635.8 ± 5.2 b	53.7 ± 0.4 b	96.9 ± 0.7 b	1324.4 ± 48.5 b
+Pulp Fiber	17.5 ± 0.3 a	556.0 ± 0.5 a	46.5 ± 0.3 a	81.3 ± 0.7 a	1019.8 ± 48.6 a

CI, color intensity; TA, total anthocyanins (mg/L); TPI, total polyphenol index; PolA (mg/L), polymeric anthocyanins; MCPT (mg/L), total tannins measured by methylcellulose precipitation method. Different letters in the same column and each type of wine mean statistically significant differences (*p* < 0.05) (n = 3).

**Table 3:** Concentration and composition of tannins of wines with and without treatment with the fining agent (pulp fiber).

Samples	TT Phloro	mDP	EGC	ECG
Monastrell				
Control	1015.5 ± 20.4 b	6.9 ± 0.2 a	612.3 ± 24.1 b	87.2 ± 7.0 b
+Pulp Fiber	850.3 ± 12.4 a	7.1 ± 0.1 a	485.9 ± 6.5 a	75.1 ± 1.1 a
Syrah				
Control	393.5 ± 7.9 b	6.4 ± 0.3 a	281.0 ± 6.8 b	80.7 ± 2.0 b
+Pulp Fiber	323.5 ± 9.9 a	6.1 ± 0.2 a	225.5 ± 6.4 a	72.9 ± 2.0 a
Cabernet Sauvignon				
Control	452.6 ± 48.3 a	6.0 ± 0.1 a	448.2 ± 52.1 a	62.5 ± 4.8 a
+Pulp Fiber	443.5 ± 50.0 a	6.7 ± 0.5 a	387.6 ± 28.9 a	61.7 ± 4.0 a

TT phloro (mg/L), total tannins measured by phloroglucinolysis; mDP, mean degree of polymerization; EGC (µM), epigallocatechin; ECG (µM), epicatechin gallate. Different letters in the same column mean, for each type of wine, statistically significant differences (*p* < 0.05) (n = 3).

**Table 4:** Concentration of histamine and OTA in the wines with and without treatment with the fining agent (pulp fiber).

Samples	Histamine	OTA
Monastrell		
Control	65.6 ± 1.2 b	7.9 ± 1.1 b
+Pulp Fiber	60.4 ± 1.0 a	4.0 ± 0.3 a
Syrah		
Control	65.2 ± 1.5 a	8.3 ± 0.1 b
+Pulp Fiber	63.0 ± 0.9 a	4.0 ± 0.2 a
Cabernet Sauvignon		
Control	66.6 ± 1.6 b	9.0 ± 0.6 b
+Pulp Fiber	61.2 ± 0.3 a	3.8 ± 0.1 a

Histamine (mg/L), concentration of histamine; OTA (μg/L), concentration of ochratoxin A. Different letters in the same column and for each type of wine mean statistically significant differences (*p* < 0.05) (n = 3).

## Data Availability

Not applicable.
